# Hospital-based interventions: a systematic review of staff-reported barriers and facilitators to implementation processes

**DOI:** 10.1186/s13012-018-0726-9

**Published:** 2018-02-23

**Authors:** Liesbeth Geerligs, Nicole M. Rankin, Heather L. Shepherd, Phyllis Butow

**Affiliations:** 1 Psycho-Oncology Co-operative Research Group (PoCoG), School of Psychology, The University of Sydney, Sydney, NSW 2006 Australia; 2 Sydney Catalyst Translational Cancer Research Centre, NHMRC Clinical Trials Centre, The University of Sydney, Chris O’Brien Lifehouse, Level 6, 119-143 Missenden Rd, Sydney, NSW 2050 Australia; 3 Cancer Council NSW, PO Box 572, Sydney, NSW 1340 Australia; 4 Centre for Medical Psychology and Evidence-based Decision-Making (CeMPED), The University of Sydney, Sydney, NSW 2006 Australia

**Keywords:** Implementation science, Health services research, Systematic review, Hospital services, Barrier analysis

## Abstract

**Background:**

Translation of evidence-based interventions into hospital systems can provide immediate and substantial benefits to patient care and outcomes, but successful implementation is often not achieved. Existing literature describes a range of barriers and facilitators to the implementation process. This systematic review identifies and explores relationships between these barriers and facilitators to highlight key domains that need to be addressed by researchers and clinicians seeking to implement hospital-based, patient-focused interventions.

**Methods:**

We searched MEDLINE, PsychInfo, Embase, Web of Science, and CINAHL using search terms focused specifically on barriers and facilitators to the implementation of patient-focused interventions in hospital settings. To be eligible, papers needed to have collected formal data (qualitative or quantitative) that specifically assessed the implementation process, as experienced by the staff involved.

**Results:**

Of 4239 papers initially retrieved, 43 papers met inclusion criteria. Staff-identified barriers and facilitators to implementation were grouped into three main domains: system, staff, and intervention. Bi-directional associations were evident between these domains, with the strongest links evident between staff and intervention.

**Conclusions:**

Researchers and health professionals engaged in designing patient-focused interventions need to consider barriers and facilitators across all three identified domains to increase the likelihood of implementation success. The interrelationships between domains are also crucial, as resources in one area can be leveraged to address barriers in others. These findings emphasize the importance of careful intervention design and pre-implementation planning in response to the specific system and staff context in order to increase likelihood of effective and sustainable implementation.

**Trial registration:**

This review was registered on the PROSPERO database: CRD42017057554 in February 2017.

**Electronic supplementary material:**

The online version of this article (10.1186/s13012-018-0726-9) contains supplementary material, which is available to authorized users.

## Introduction

Health service interventions that are effectively implemented are associated with improved patient and staff outcomes and increased cost-effectiveness of care [1]. However, despite sound theoretical basis and empirical support, many interventions do not produce real-world change, as few are successfully implemented [2, 3], and fewer still are sustained long-term [4]. The ramifications of failed implementation efforts can be serious and far-reaching; the additional workload required by implementation efforts can add significant staff burden [3], which can reduce the quality of patient care and may even impact treatment efficacy if interventions disrupt workflow [5]. Additionally, staff who bear the burden of implementing new interventions may be reluctant to try alternatives if their first experience was unsuccessful [6]. A thorough understanding of the barriers and facilitators to implementation, as well as an ongoing assessment of the process of implementation, is therefore crucial to increase the likelihood that the process of change is smooth, sustainable, and cost-effective.

Implementation science focuses on factors that promote the systematic uptake of research findings and evidence-based practices into routine care [7]. A number of frameworks have been developed to describe and facilitate this process and can be classified into three main groups with the following aims: describing or guiding the process of translating research into practice (process models), understanding and/or explaining what influences implementation outcomes (determinant frameworks, classic theories and implementation theories), and evaluating implementation (evaluation frameworks) [8]. As our review seeks to recognize the specific types of determinants that act as barriers and facilitators, we drew mostly from determinant frameworks such as the Promoting Action Research in Health Services (PARiHS) framework [9] and the Consolidated Framework for Implementing Research (CFIR) [10]. The PARiHS highlights the importance of evidence, context, and facilitation [9], while the CFIR proposes five key domains of influence: inner and outer setting, individual characteristics, intervention characteristics, and processes [10]. The focus of such frameworks is on understanding and/or explaining influences on implementation outcomes, and they are therefore often used by researchers and clinicians to plan their implementation, develop strategies to overcome barriers, and support successful delivery.

However, research in the field has also been impeded by the use of inconsistent language and inadequate descriptions of implementation strategies [11], an issue that has recently been addressed by the development of the Expert Recommendations for Implementing Change, which has resulted in a refined compilation of strategy terms and definitions [12]. In addition, recent reviews of commonly used strategies, such as nominating intervention champions, have found that they are not uniformly successful [13], suggesting that such approaches are not “one size fits all” and must instead be selected in line with the context and needs of the population. Therefore, there has been an increasing call to explore implementation frameworks by systematic review, in ways that not only identify barriers and facilitators but seek to explore the mechanisms underlying change, and the processes by which these barriers and facilitators relate to each other and to implementation success outcomes [3, 14] in the specific context in which they are trialed.

Hospitals are one such specific context, with unique populations, processes, and microsystems, which may encounter unique barriers [15]. Additionally, interventions within hospitals are often complex and multi-faceted and must contend with barriers across a wide range of settings. While systematic reviews have focused on the hospital context as regards integrated care pathways [16], no systematic review to date has focused on the implementation of patient-focused interventions in the hospital setting.

The current systematic review therefore had two key aims: first, to identify staff-reported barriers and facilitators to implementation of patient-focused interventions within the hospital context, and second, to define and explore relationships between these, in order to generate practical strategies that can assist tailoring to individual service needs. We also sought to explore the fit between existing frameworks and components of real-world implementation studies, to contribute to the growing evidence base for these frameworks and to identify those likely to be of most use to clinicians, researchers, and administrators in designing and conducting implementation studies.

## Methods

### Registration

This systematic review is registered on PROSPERO (17.02.17, registration:2017057554) [17].

### Search strategy

A search of the relevant databases (Psych Info, MEDLINE, PubMed, Embase, CINAHL, and Web of Science) was conducted, with results limited to articles published up until 31 December 2016. A comprehensive list of search terms (see Additional file 1) was developed based on the terminology of the field and keyword lists of relevant papers (see summary in Table 1). Keywords that mapped to specific Medical Subject Headings for each database were selected to ensure an inclusive search approach. Returned search results were screened for duplicates. Ethical approval was not required for this review.

**Table 1:** Summary of database search terms

Process	[implementation$ or dissemination$ or roll-out or knowledge translation or knowledge transfer] AND
Type of change	[intervention$ or treatment plan$ or care plan or pathway$] AND
Population/setting	[health care or health care service$ or health care utilization or health care delivery or hospital services or health services research or clinical service$ or hospital program$ or tertiary service or hospital] AND
Mechanisms	[facilitat$ or barrier$ or challenges or barrier analysis or process analysis or enabl$ or change agent] AND
Intervention type	[psychological or psychosocial or psychology]

### Eligibility criteria

A checklist of inclusion and exclusion criteria was developed to guide selection of appropriate studies (Table 2). During this process, all authors reviewed a sub-sample of articles (10%) to refine inclusion and exclusion criteria and ensure criteria could be consistently applied.

**Table 2:** Inclusion and exclusion criteria

1. Types of studies	Quantitative or qualitative original studies published in full including:
	- Interviews/focus groups
	- Surveys/questionnaires
	Exclusions: Review papers, editorials, commentary/discussion papers, papers published in languages other than English, conference posters or oral presentations not available in full text, book chapters.
2. Study settings	Hospital settings including:
	- Inpatient
	- Outpatient hospital settings where implementation is based in the hospital context
	- Mixed context studies where at least one setting is hospital-based (and data is reported for staff in that setting)
	Exclusions: community-based, population-based, school-based, prison-based, outreach studies, nursing homes.
3. Population	Hospital staff of any type including:
	- Health care providers (doctors, nurses, allied health professionals), IT, managers, administrators
	Exclusions: no staff who were working in the hospital at the time of implementation were excluded. Any papers that collected data from staff who were not hospital-based were excluded based on criterion 2, study setting. For example, studies based in community health settings with community health workers were excluded based on setting. However, if a hospital study involved both clinical and community staff in a hospital-based implementation, all staff involved in the implementation were included.
4. Interventions	The intervention focused on direct patient care outcomes including:
	- Direct patient interventions such as therapy or behavioral change interventions
	- Interventions with direct patient benefit, e.g., hygienic interventions, staff behavioral or communication based interventions designed to improve patient outcomes
	Exclusions: medical record management or IT interventions, interventions focused on administration outcomes, e.g., rostering change interventions.
5. Formal collection of data about implementation processes	The study contains formal, objectively collected data (quantitative or qualitative) from staff on barriers and facilitators to implementation (at any stage: pre, post, or during the process) including:
	- Interviews/focus groups with staff participants where questions specifically asked about the implementation
	- Surveys/questionnaires with staff participants on barriers to the implementation
	Exclusions: any papers that did not directly assess the implementation process, as well as any studies that did not provide any formal data (as specified above) from staff participants about the implementation process. Therefore all studies that assessed the intervention only were excluded, as well as studies which provided only descriptive or anecdotal information about the implementation.

A study was eligible for inclusion if (1) it was an original research study published in full, (2) it was hospital-based, (3) participants surveyed about the implementation were hospital staff, (4) the intervention involved direct patient care, and (5) it included formal collection of data from participating staff about barriers and facilitators to the implementation process.

No study design was excluded, but studies needed to meet all five criteria to be eligible. Only studies in English were assessed, and studies that could not be accessed in full (such as conference abstracts) were excluded, as there was insufficient detail to determine whether they met the additional exclusion criteria. We included studies that provided any formal data, quantitative (such as surveys and Likert ratings) or qualitative (such as interviews and focus groups), regarding implementation barriers and facilitators either anticipated pre-implementation or encountered during implementation. In assessing eligibility, included studies were required to have collected formal data related to the implementation specifically, rather than the intervention itself [11]. The need to separate assessment of implementation processes from interventions has been highlighted in the recent Standards for Reporting Implementation Studies (StaRI), which note that this distinction is crucial for allowing researchers to identify the key components that lead to effective translation of evidence into practice [18]. Therefore, our analysis focused solely on papers which identified the barriers and facilitators that affect the implementation process, rather than the intervention. This meant that all papers that reported only data about the intervention outcomes (including effectiveness data) were not considered eligible. Interventions were defined as being focused on patient care if they had either direct patient contact (such as patient-targeted behavioral interventions) or had a direct impact on patient outcomes (such as quality and safety interventions). Some studies retrieved dealt exclusively with introducing electronic records; these were not included as they had no patient-centered focus. Further detail on exclusion and examples of excluded papers for each eligibility criterion are provided in Additional file 2.

Several theories and taxonomies have been proposed to guide measurement of success that include issues of uptake, penetration, cost-effectiveness, and sustainability [19]. However, very few identified studies used a theory or framework to guide their definition of success. Therefore, for the purposes of this review, we used the barometer of success defined by each individual study.

### Study selection process

Decisions regarding eligibility were made by LG and verified by co-authors. Studies were initially screened by title and abstract; the remaining articles underwent a full-text analysis. All studies were initially reviewed by the first author (LG), with a subset of articles (10%) also subject to team review to assure consistency. No formal analysis of agreement was carried out for this stage of study selection, as any disagreements were resolved by iterative discussion until consensus was reached.

### Data extraction and analysis of included articles

For all included articles, we collected descriptive information comprising author, date of publication, participant group, and study design. To extract and synthesize data on barriers and facilitators, we used the Framework Analysis approach [20] and generated a data abstraction matrix to organize and display content.

Qualitative synthesis was accomplished in a series of stages as follows: (1) reviewing a subset of the included articles to familiarize the research team with the literature base, (2) deriving a series of codes and subcodes that reflected key concepts within the data, (3) developing these concepts into an overarching thematic framework of categories, (4) systematically indexing each article according to the framework, entering summary data (quantitative studies), and verbatim quotes (qualitative studies) into the cells of the matrix. Initial codes were generated by the first author and were refined together by the team in a series of iterative reviews, to ensure clarity and synthesis of data [21].

Given the unique context being explored, we decided to undertake this inductive approach rather than using an existing theoretical framework initially, as this allowed us to see what factors arose in real world studies, rather than imposing a specific framework initially.

### Quality assessment

We used the Critical Appraisal Skills Program (CASP) [22] for qualitative studies, and the Mixed Method Assessment tool (MMAT) [23] for quantitative and mixed method studies. These were selected because they have an extensive scoring guide, sound psychometric properties, capture a range of key components of qualitative research (CASP), and specifically assess both quantitative descriptive and mixed methods research (MMAT).

Quality assessment was based on the implementation data provided, rather than the overall study data. All papers were reviewed against these checklists (LG), and a subset of papers (6) were reviewed by a second author (NR) to assess for agreement. We defined agreement as the proportion of items where both raters gave a positive (yes) or a negative (cannot tell, no) score. A formal analysis of agreement was carried out based on Cohen’s Kappa for inter-rater reliability, and scores varied from 0.45 to 0.61 between raters, indicating moderate to substantial agreement according to Landis and Koch’s standards [24]. Discrepancies were resolved through iterative discussions.

## Results

### Included studies

**Fig. 1:**
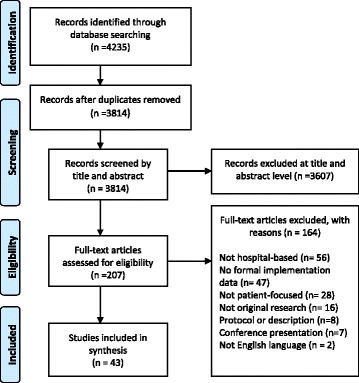
PRISMA flow diagram of study selection process. Some papers were excluded on more than one criterion, therefore total excluded *N* > 3684

Of the 4239 articles identified, 43 met the inclusion criteria (see Fig. 1). Study characteristics are reported in Additional file 3.

### Study characteristics

#### Study origin

Studies were largely based in developed countries, including the USA (12), the UK (8), Canada (6), Australia/New Zealand (6), Denmark (2), Sweden (1), Finland (1), Italy (1), and the Netherlands (1). The remaining studies originated in Uganda (1), South Africa (1), Tanzania (1), Ghana (1), and Mexico (1).

#### Study designs

Studies were predominantly cross-sectional (*n* = 41) designs, with only two using a longitudinal design.

#### Participants

Participant response reporting varied as some interventions were carried out at the macro-level (e.g., across several hospitals) and some at the micro-level (e.g., a pilot in a single ward). Some studies reported exact numbers (*n* = 2 to 132) while others only included the number of hospitals participating (*n* = 1 to 38). Participant type was also reported inconsistently, with some studies specifying only that interviews were carried out with “project participants,” while others specified respondent type (e.g., nurses, clinical specialists, allied health professionals, and administrators).

#### Methods

The majority (*n* = 37) of studies used qualitative methods exclusively, three used mixed methods, and three quantitative methods exclusively. Semi-structured interviews were the most common data collection strategy (in both qualitative and mixed methods) followed by focus groups, audit, and observation. Quantitative and mixed methods studies used questionnaires (designed for the study) or validated measures.

#### Types of implementation

There was great variation in the implementation of interventions and the health states targeted, as shown in Tables 3 and 4.

**Table 3:** Population health states targeted in included studies

Health state	Included studies
Mental illness	7
Pregnancy/neonatal	7
General population	6
Oncology	5
ED	4
HIV	3
Pediatric	2
Palliative	2
Geriatric care	2
ICU	1
Bereaved parents	1
Congenital heart failure	1
Speciality areas (orthopedics, cardiology, urology, women’s health, general surgery, neurosurgery)	1
Traumatic injury	1

**Table 4:** Intervention approach in included studies

Intervention approach	Included studies
Supportive or behavior change intervention/clinic	16
Screening/assessment tool/process	10
Clinical or care pathway/guidelines	7
Medical procedure	3
Safety and quality	3
Breast feeding/infant care	2
Reporting system	1
Patient decision aids	1

#### Explicit use of conceptual theory or framework

Less than half the studies (*n* = 16) reported using theory to guide their implementation, most commonly the Theoretical Domains Framework, the PARiHS framework, the Realist Evaluation framework, and the Contingency Model.

#### Reporting of barriers and facilitators

Most studies focused explicitly on barriers and facilitators to implementation (*n* = 28), the remaining 15 studies reporting barriers and facilitators as secondary data (with a primary focus on effectiveness or outcomes of the intervention).

#### Study quality

Studies focusing on implementation processes often had a quality improvement or action research focus that did not clearly align with any of the major checklists and therefore failed to address some criteria. Where implementation data about barriers and facilitators was a secondary focus, reporting on these issues was of lower quality, despite overall high-quality reporting on other outcomes. Areas of poorer quality included a lack of detail on data collection methods, participants, response rates, and representativeness (Table 5). Few researchers discussed reflexivity, despite increasing recognition that research teams are likely to affect implementation processes [25, 26].

**Table 5:** Quality checklist criteria

Quality checklist criteria	Included studies that met this criteria (rating yes)
Critical Appraisal Skills Program (CASP)	(*N* = 37)
1. Was there a clear statement of the aims of the research?	35/37
2. Is a qualitative methodology appropriate?	37/37
3. Was the research design appropriate to address the aims of the research?	33/37
4. Was the recruitment strategy appropriate to the aims of the research?	30/37
5. Was the data collected in a way that addressed the research issue?	32/37
6. Has the relationship between researcher and participants been adequately considered?	2/37
7. Have ethical issues been taken into consideration?	34/37
8. Was the data analysis sufficiently rigorous?	31/37
9. Is there a clear statement of findings?	34/37
10. How valuable is the research? (no rating)	Rating not indicated for this item
Mixed Methods Appraisal Tool (MMAT)	(N = 3)
Are there clear qualitative and quantitative research questions (or objectives), or a clear mixed methods question (or objective)?	3/3
Do the collected data allow address the research question (objective)? E.g., consider whether the follow-up period is long enough for the outcome to occur (for longitudinal studies or study components).	2/3
1.1. Are the sources of qualitative data (archives, documents, informants, observations) relevant to address the research question (objective)?	2/3
1.2. Is the process for analyzing qualitative data relevant to address the research question (objective)?	1/3
1.3. Is appropriate consideration given to how findings relate to the context, e.g., the setting, in which the data were collected?	2/3
1.4. Is appropriate consideration given to how findings relate to researchers’ influence, e.g., through their interactions with participants?	0/3
4.1. Is the sampling strategy relevant to address the quantitative research question (quantitative aspect of the mixed methods question)?	1/3
4.2. Is the sample representative of the population understudy?	1/3
4.3. Are measurements appropriate (clear origin, or validity known, or standard instrument)?	1/3
4.4. Is there an acceptable response rate (60% or above)?	1/3
5.1. Is the mixed methods research design relevant to address the qualitative and quantitative research questions (or objectives), or the qualitative and quantitative aspects of the mixed methods question (or objective)?	3/3
5.2. Is the integration of qualitative and quantitative data (or results) relevant to address the research question (objective)?	2/3
5.3. Is appropriate consideration given to the limitations associated with this integration, e.g., the divergence of qualitative and quantitative data (or results) in a triangulation design?	1/3
Mixed Methods Appraisal Tool (MMAT; Quantitative descriptive)	(*N* = 3)
Are there clear qualitative and quantitative research questions (or objectives), or a clear mixed methods question (or objective)?	3/3
Do the collected data allow address the research question (objective)? E.g., consider whether the follow-up period is long enough for the outcome to occur (for longitudinal studies or study components).	3/3
4.1. Is the sampling strategy relevant to address the quantitative research question (quantitative aspect of the mixed methods question)?	3/3
4.2. Is the sample representative of the population understudy?	2/3
4.3. Are measurements appropriate (clear origin, or validity known, or standard instrument)?	2/3
4.4. Is there an acceptable response rate (60% or above)?	2/3

### Key findings of barriers and facilitators to implementation

Qualitative synthesis identified 12 distinct categories of barriers or facilitators, which were grouped into three main domains: system, staff, and intervention. Each domain was associated with clear sub-domains, as shown in Table 6. The detail about each domain is presented, with illustrative quotes, in Table 7.

**Table 6:** Identified barriers and facilitators to implementation

Domain	Sub-domain	Brief description	Number of included studies citing barriers or facilitators in this domain
System			
Environmental context	IT, trial staff, time, workload, workflow, competing trials, space, movement and staff turnover	The physical, structural resources of the context, along with its processes and personal resources	37
Culture	Attitude to change (readiness and agents), commitment and motivation, flexibility of roles/trust, champions/role models	The system culture, beliefs and behaviors in relation to change and staffing roles	28
Communication processes	Processes within the context	The processes of conveying information within the system, in terms of both online and in-person methods	25
External requirements	Reporting, standards, guidelines	Any external pressures or expectations that impact on the deliverables of the system	4
Staff			
Staff commitment and attitudes	Perceived validity/need, ownership, perceived efficiency, perceived safety, belief in change/readiness for change	The micro-level beliefs, attitudes and behaviors toward change in general, and the intervention specifically	33
Understanding/awareness	Of the goals of the intervention, and of the processes/mechanics	Understanding of the aims and methodology of the intervention	22
Role identity	Flexibility, responsibility	Beliefs and attitudes towards one’s work role and responsibilities	13
Skills, ability, confidence	To engage patients and overcome patient barriers, to carry out the intervention, to manage stress/competing priorities	Staff sense of their capacity to carry out the tasks of the intervention, while managing the barriers posed by the target population and their work environment	30
Intervention			
Ease of integration	Complexity, cost and resources required, flexibility (to respond to patient, staff and system), acceptability/suitability to system, staff and patients; fit for context	How well the intervention “fits” with the current system, resources and needs of the population and context, as well as its ability to adapt and respond when changes are needed	30
Face validity/evidence base	Theory and evidence	The extent to which the intervention is grounded in solid evidence regarding a known issue, and how effective it looks to be in terms of meeting its aims	12
Safety/legal/ethical concerns	Patient or staff safety; medico-legal concerns	How well an intervention addresses important issues of safety and legality to protect staff and patients	6
Supportive components	Education/training provided, marketing/awareness, audit/feedback, involvement of end users	The components of the intervention which work to support and facilitate the changes necessary	38

**Table 7:** Identified domains and quotes from included studies

Factor	Illustrative quotes
System	
Environmental context	Workload: “The difficulty is not actually doing the observation, it’s …having the time to go and write it down, and then talk to somebody about it” (Ward co-ordinator) [27] Availability: “It’s not always easy depending on the staffing levels on the ward. Obviously, if you’ve got a lot off sick or on annual leave or whatever, the numbers are short, it’s not always possible….”(Ward co-ordinator) [27] Burden falls on small number of staff: “I tried to leave [POS] questionnaires for people in the diary and it just didn’t work. I actually came in [on days off] to do it, because I rang up to see if anyone had bothered and they hadn’t” [31] Need for institution level support: “There needs to be explicit support from the institution that spending time on these issues is time well spent. That it’s valued and supported, … and that it is a priority (Psychiatrist)” [6] Physical space: “There are too many people for too little space, especially for people who are only going to watch.” [41] Workflow systems: “[We] need to address the hospital management so that they can revise the system of allocating…who is the responsible team even on the weekend. (Physician)” [5] IT: “(we need the)…ability to track referrals and see whether the patient actually saw the psycho-oncologist because it doesn’t always happen…and to have that in some sort of standardized, accessible way, ideally as part of the medical record.” (Medical oncologist) [6] System level: “support should be at the system level in terms of how it’s integrated, in routine documentation, in IT systems and in quality review.” (Nurse clinician-researcher) [6]
Culture	Attitude toward change: “Sometimes it seems a very big mountain’; it’s going to take a while to change”(Focus Group) [37] System level commitment: “My coworkers are flexible and even double their workload so you can talk with the parents in peace, it’s considered such an important thing” [40] Role flexibility: “Doctors have their title and so they think that no one else knows anything. . . . They are going to be hostile [towards us]” [41] Staff role: “I don’t mind [having the role of ward coordinator]. I’m the infection control link nurse, so I see it as part of that role really, hand hygiene…” (Ward coordinator) [27] Champions: “I did find sometimes [as a consequence of delivering the intervention], people in groups was like against me [.. .] they try to find another problem of me and go talk to the manager regarding that... because I pick them up on their problem they’re going to talk to the manager” (Ward coordinator) [27]
Communication processes	Lack of interdepartmental communication: “Developing this program requires so much collaboration between so many different departments–I don’t know if it happens all the time or all that easily.… it’s tough to have a communication system between departments and across systems–e-mail and access to patient information is not always smooth” [67] Culture of open communication: “We have a new administration that promotes a very openness in communication, and is very quick to recognize systems problems and not people problems, so to speak” [44]
External requirements	“If you have no accreditation then you don’t get reimbursed and you don’t stay open.” [44] “So we wrote the policy to be a mandatory directive so that those people at the ground level had the topdown support. To be able to say we have been told we have to do this, so you (hospital management) need to support us” (Focus group) [37] “And if… you’ve got senior buy-in to say ‘this is an expectation of our cancer services… if you provide the support underneath that and the resourcing of the implementation to a certain degree, you’re kind of covering both ends” (Nurse) [6]
Staff	
Staff commitment and attitudes	Attitude toward the intervention: “the cardiologists say they don’t need it, they know what to do with these patients” [45] Beliefs regarding need for intervention: “if we’re able to communicate the difference that this has the potential to make to women in their care, they’re far more likely to champion it…” [28] Motivation: “They may feel that they’re losing control or that they’re being forced to do something” [45] “I’m really very passionate about this [the intervention] that we’re doing, so I’m really striving to do it” (Ward coordinator) [27] Ownership:“…getting engagement with psychosocial services and the nursing staff… is really important because the bottom line is that at the end of the day they’re going to implement it” (Nurse) [6]
Understanding/awareness	“I still feel that there’s a view out there that it’s…a fanatical way of operating” (Focus Group) [37]
Role identity	“(there is) …a lack of clarity about who’s role it is, who the decision maker is… It’s not that uncommon that someone says ‘well that’s my role’ and everyone in the rest of the team goes ‘is it?’” (Nurse) [6] “I think it’s everybody’s responsibility you know. . .Just getting everybody involved rather than a few motivated members of the team who are interested in it” (Nurse) [47]
Skills, ability, confidence	Confidence: “I do not have the confidence to work with a doctor.” (Traditional Midwife) [41] Skill: “I felt that if I disturbed something while I was talking to them, I don’t have the psychological back up for them” [31]. Patient-related barriers: “some of the patients are so very rude. Angry and rude. You won’t even be able to approach the to ask them questions” [35] Time management: “I’ve felt stressed in terms of, I’ve got to get it done and, you know, the clock’s ticking and I’ve got other things to do” (Ward coordinator) [27] Competing demands: “Social workers have too many clients to add positive prevention to their caseloads. The workload was unmanageable” [32]
Intervention	
Ease of integration	Multiple stages of intervention: “me in the unit telling them “there’s a counselor that you have to come and see tomorrow”, there’s no way he’s coming back” [35] Simplicity: “Just looking at the ten steps... it is achievable” [37] Resources and workload: *“*We were getting a large number of phone calls…and it was easier, frankly, to do what we’ve been doing …and not have to put up with numerous calls” [45] “It became time consuming, with the end result being the same” [45] Suitability and fit“. . . it’s a part of your routine already so I don’t find it difficult, it’s just finding ways of how to do it, I mean it’s not too difficult” [49] Acceptability to staff: “Clinical pathways are used in lots of different areas and the ease at which it is to implement these things is a challenge and… (there is a) degree of fatigue around different things that get implemented… particularly once you get down to department level” (Nurse) [6] Fit for patient populations: “we focus a great deal on changing clinicians’ expectations and skills, but I don’t think we’ve even tackled too closely an understanding of what’s needed in order to make services more acceptable to patients.” (Psychiatrist) [6]
Face validity/evidence base	Evidence: “I feel there has to be overwhelming evidence of the benefits in using it and also some kind of reassurance in the evidence that using the i.v. component wasn’t going to have a negative impact in terms of development of resistance” [49] Awareness: “I think there is certainly plenty of evidence there that some of us should be looking at and I think the big problem is . .not everybody has fully appraised the papers” [49]
Safety/legal/ethical concerns	Safety: “Sometimes I feel a little bit worried that, have I given them the right advice. . . the right advice I should be giving them” (Allied Health professional) [47] Responsibility: “I would not have so much responsibility. Any complications would be the responsibility of the doctor”(Traditional midwife) [41] Ethics: “I don’t like having my name attached to it in some way by endorsing it. By giving it to the patient I’m endorsing its content …. That makes me feel uncomfortable” [53] Liability: “I think that is a part of our culture, when people feel very protective and somewhat defensive because they are concerned about sitting on a witness stand, or being sued, or having some risk” [44]
Supportive components	Training: “We are getting new doctors especially interns every time. Updating when new information arises or when changing protocols happens is very important for proper care of patients. (Nurse)” [5] Repetition: “It’s not just the education getting them past the bad habits, you have to keep going back and back and repeating and then they get into a rhythm . . . they need constant reinforcement” [44] Professional support: “We don’t receive clinical supervision at all and when you call them after months and try to recollect the child’s death… it would give me strength to provide more phone calls and to invest in this program” [40] Audit and feedback: “Anytime you’re monitoring something, compliance is better . . . everyone is willing to change…it’s just a habit and habits are hard to break” [44] Evidence of outcomes: “Someone needs to show that this will actually lead to not necessarily a substantial increase in referrals to the high end of the services, but actually a better utilization of those resources.” (Nurse) [6] End user involvement:“…people need to feel that this is an important priority, that they’re involved in shaping it, localizing it, customizing it, that it reflects what they can do and achieve, that they’re supported in it”(Psychiatrist) [6]

### System level barriers and facilitators

#### Environmental context

Barriers directly related to the hospital environment included workload and workflow, physical structure, and resources. Staff workload and lack of time for implementation were the most commonly cited barriers [27–29]. Staff shortages, high staff turnover, or changes in roster compounded this issue [30], resulting in burden for implementation falling on small numbers of staff who were most interested, rather than generating change at the institution level [31]. Several studies targeted this issue by hiring additional staff, such as a research coordinator [32], or delegating parts of the intervention to the research team. However, this was dependent on research team capacity and funds; sustainability of these strategies after the research team left was not addressed [32]. In contrast, support provided at the institutional level for staff to have time for implementation was believed to be a more sustainable facilitator [6].

Implementation processes were also stymied by systemic workflow organization and staff movement [33]. Hospital workflow around division of responsibilities, transfer of work between shift-working staff, and systems of care governing how and when patients were seen during changeover periods often resulted in inconsistent implementation or significant gaps in the process [5]. Movement of staff across multiple roles or areas of the site resulted in decreased knowledge and movement of patients made consistency in the implementation process challenging [34].

The physical structure of the hospital site created barriers to implementation, such as lack of private space for interventions requiring sensitive discussion [35, 36]. Implementation involving IT innovations often faced barriers related to the hospital’s ability to accommodate new systems [6]. A final barrier was the popularity of interventions in hospital wards, which results in staff reporting fatigue toward new initiatives [6] or feelings of tension when juggling hospital priorities alongside intervention goals [37, 38].

#### Culture

Barriers related to workplace culture centered around system-level commitment and change readiness. Low levels of commitment often occurred in response to structural changes, such as high turnover, which left staff feeling demoralized and unable to accept additional challenges required by implementing the intervention [30]. Support from management regarding the importance of change and organization-level commitment to new processes was crucial to combating this [38–40]. Several interventions also used champions or coordinators to facilitate motivation [39], although some staff reported experiencing negativity from colleagues as a barrier to carrying out this role effectively [27].

Workplace culture barriers also included the level of role flexibility and trust between different clinicians involved in the intervention. Congruence between the intervention requirements and staff roles was important [27]. Staff who reported that implementation required them to carry out duties beyond their role reported struggling, especially if they anticipated judgment from colleagues [41]. However, other respondents felt that building trust across the team could address these concerns [41].

#### Communication processes

The efficacy of communication processes emerged as the third system-level factor, particularly where interventions required collaboration between staff of different disciplines [20, 42]. Lack of interdepartmental collaboration, miscommunication, and fragmentation between practitioners could serve as a significant barrier to successful implementation [28, 43]. Study environments that promoted open and clear communication motivated staff to take on challenges, and feel safe about reporting errors or issues, resulting in more successful implementation [44].

#### External requirements

The final system-level domain related to external pressures such as pending audits, accreditation requirements, or assessments by an external body. These were strong influencers of motivation and commitment to the intervention [44], particularly if perceived as contributing to better institutional outcomes. The perception of external obligations alone was considered a source of motivation as it encouraged management support for staff who were trying to implement the intervention [37]. Participants noted that implementation as part of hospital policy or standards were a strong facilitator to lasting change [6].

### Staff level barriers and facilitators

#### Staff commitment and attitudes

While system domains focused on the overall structure and culture, staff domains were more focused on the individual, and the experiences, motivations and beliefs of those staff directly involved with carrying out the intervention. Commitment and motivation was identified as the first staff-level barrier, and this was clearly influenced by staff attitudes regarding the proposed intervention, which directly impacted their engagement with the implementation process. In some instances, participants questioned intervention validity, for example, whether patients would respond honestly to screening [31] and whether the intervention would have any real effect on behavioral change [43]. Lack of belief in the intervention was associated with variability in adherence to intervention guidelines, causing a barrier to successful implementation [34]. Equally, if staff felt they were already equipped to address the issue targeted by the intervention, they were less likely to adopt the changes required to achieve full implementation [45].

Change readiness levels of individual staff also influenced commitment; even in cases where the overall culture was positive, individual clinicians were not always responsive to new ways of doing things, in part due to feelings of losing control in their role, or feeling that they were forced to make changes [45]. To combat this, several studies noted the impact of sharing informal intervention “success stories” in shifting staff morale and openness to change [32, 46]. A sense of ownership, and a belief in the process, was another key facilitator and was more likely to occur when staff felt engaged in the process of implementation [6, 28].

#### Understanding and awareness

Staff knowledge of the aims and process of the intervention was key to ensuring effective implementation. Misinterpretation of the intentions or meaning of interventions could trigger unnecessary resistance toward the implementation [37]. Confusion or disregard of intervention processes could also impact implementation, as it meant that staff did not follow procedure [35]. In some instances, this lack of awareness was addressed via additional training and education [34, 37]. Where an intervention did require additional work or resources, it was important that staff understood that it would lead to longer term positive outcomes and reduction in overall burden [38, 45].

#### Role identity

Motivation to adopt changes required for implementation was often decreased when staff felt the intervention was not part of their role (22) or experienced confusion regarding who should fulfill the role [6]. Where interventions called for staff to go beyond their previous role, this could also create resistance or hesitation [32]. However, role responsibility was likely to be increased in situations where participants felt a sense of duty or obligation to the intervention [47].

#### Skills, abilities, and confidence

In cases where the intervention required staff to implement a new approach, lack of confidence or ability proved a significant barrier, with staff who reported lower skills expressing greater resistance to the implementation [31, 41]. Participants at times felt ill-equipped to carry out the tasks of the intervention, particularly if it required skills in an area they felt they had not been trained for [31]. Participants also felt under-resourced or unable to overcome a range of patient-related barriers to the intervention such as engaging challenging populations on difficult topics (e.g., substance use) [35]. Participants who felt they had the skills to engage and build rapport with patients described this ability as a facilitator to change [31]. Ability to carry out the intervention was further impacted by stress and time management challenges [27]. Participants at times reported that their level of responsibility was unmanageable [32], expressing concerns about the potential of burnout [29], or that the physical care of the patients needed to be prioritized over the implementation [38, 48]. However, where an intervention lead to greater consistency of practice, this was reported as a facilitator, leading to increased ability and decreased stress overall [41].

### Intervention level barriers and facilitators

#### Ease of integration

Interventions that fitted the existing hospital system and ways of working were more likely to be reported as successful [49], while interventions that required change to standard processes were more likely to report delays and gaps in implementation processes [50]. However, these issues could be overcome in interventions that were flexible and iterative, such as those that engaged in ongoing tailoring and review [50]. The use of action research methods and frameworks facilitated this process, enabling researchers to respond to concerns and allowed timely intervention amendments to be made [34].

Intervention complexity often made integration more challenging. Where interventions required new operating systems, IT functionality and accessibility issues were commonly reported [51, 52]. Complexity also related to intervention design: interventions that involved multiple health professionals across a range of contexts increased the likelihood of delays and miscommunications [49]. Similarly, interventions involving additional forms or screening tools created extra work for staff, and more errors in process were likely. This issue could often be targeted by simplifying forms and tools to make the process more user-friendly [34, 50]. Interventions that were perceived as simple and accessible were more likely to receive positive endorsement and greater engagement with the implementation process [37].

Acceptability and suitability of an intervention to system, staff, and patient influenced how easily it was integrated. Sometimes, the intervention did not suit the system, requiring staff to seek out patients normally seen in a different part of the hospital [35]. Staff sometimes identified a particular intervention was better suited to a different setting, where greater needs existed [45]. The cost and resources required by an intervention, in terms of work, time and stress, also influenced acceptability, and were often cited as reasons for withdrawing from, or having negative feelings toward, the implementation process [45]. Finally, acceptability of the intervention to the patient was key to integration; staff encountered barriers where patients perceived that the intervention was not relevant, such as in the case of lifestyle change interventions [47] or screening for problem drinking [35]. Patient populations were often highly complex and did not suit the straightforward pathways or interventions proposed [45]. Staff highlighted the importance of considering this in the pre-implementation design phase [6].

#### Face validity and evidence base

Many participants expressed concerns about the evidence base of interventions, and this was frequently cited as a barrier to implementation. Communicating and making the evidence accessible to staff in was considered a key facilitator as lack of awareness of the evidence was commonly reported [49]. When participants felt confident about the evidence and the intervention rationale, this increased motivation to support the implementation overall [6, 28].

#### Safety, legal, and ethical concerns

Many participants raised concerns about intervention safety, particularly where change of care was required. Participants raised this as a barrier when they were asked to deliver information they did not agree with [53]. Conversely, an intervention perceived as leading to potentially decreased risks and improved care was seen as a facilitator [41]. Ethical issues concerning patient well-being and patient confidentiality were sometimes raised. For example, when interventions required shared platforms, participants noted that confidentiality relating to user privacy needed to be considered and that patient awareness of the shared platform could influence information disclosed [51]. Concerns regarding legality and fear of litigation were also commonly cited barriers when interventions called for changes in roles and responsibilities [44, 54]. Concerns about safety meant that staff were less likely to endorse or fully participate in the implementation [53].

#### Supportive components

Training, awareness raising, audit/feedback, and engagement with end users could all serve as barriers or facilitators. Lack of training and awareness of intervention processes was seen as a key barrier, and in cases where staff turnover was high, regular in-services were noted as crucial to facilitate implementation [5]. Repeated training and awareness campaigns were seen as necessary to reinforce new processes and behavioral patterns [44], although access and time to attend training, along with availability of professional support, were common challenges [40]. These awareness-raising activities were perceived as most useful when they highlighted the evidence and need for the intervention, as well as the likely benefits to staff and patients [6].

The importance of regular audit, such as real-time monitoring of admissions to ensure fidelity, was also reported as helpful to the implementation success [55]. These strategies were also associated with improved motivation and demonstrated the utility of the intervention [28, 44]. Finally, participants highlighted the importance of engaging with the intervention end users (i.e., themselves and their colleagues) to facilitate the process of implementation in a way that was acceptable, appropriate, and sustainable [6]. Studies which had adopted models of iterative implementation, such as participatory action research, reported greater engagement from end users [34].

### Reported frequency of barriers and facilitators

The number of studies reporting barriers and facilitators for each domain are shown in Table 6. The most commonly reported domains impacting implementation success were environmental barriers at the system level, staff commitment, and attitudes toward the intervention at the staff level and supportive components at the intervention level. We note that these are only the most commonly reported barriers, which does not indicate that they are the most critical or important. However, it does convey a sense of those issues most likely to occur in the hospital setting, when carrying out patient-focused interventions.

### Links and relationships between domains

**Fig. 2:**
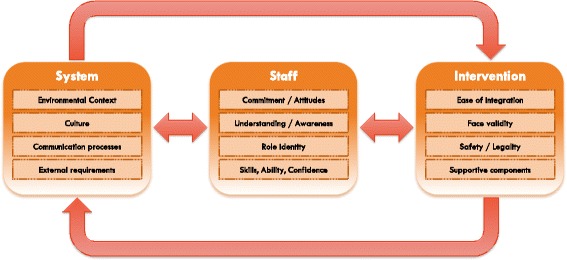
Bi-directional associations between key domains

In addition to the above domains influencing implementation success directly, associations between domains were also identified, in which facilitators from one domain were able to impact barriers in other domains (Fig. 2). This occurred most clearly at the staff level, which was easily responsive to intervention level barriers, and also highly susceptible to changes at the system level. This association was reciprocal, with staff barriers shaping elements of the intervention itself, particularly where the intervention was responsive to end user involvement [34, 48]. Staff could also impact system level barriers, providing feedback that led to changes in organizational culture and communication processes.

Intervention domains were also responsive system domains, particularly in times of deficiency, when the environment lacked concrete resources or a supportive workplace culture. Interventions would strive to address this by increasing their internal support (via additional staff or engagement meetings) [32] and ensuring ease of integration (by flexibly altering intervention components where possible) [50]. Similarly, system domains could raise significant barriers if the intervention had not foreseen and addressed them or did not have the ability to respond flexibly. This was noted in cases where hospitals underwent staffing changes, renovations, or procedural changes, which meant the intervention could not proceed as anticipated or could not be sustained [30, 34].

Associations also appeared to move in cycles, where the system might influence the staff, which in turn influenced the intervention, which in response sought to influence the system. Thus, the process was continually dynamic and iterative, explaining why interventions could fail for many different reasons, even with the best grounding in theory and planning. Our findings suggest that implementation success is not simply about selecting and delivering strategies but about reflexive awareness of emergent influences that arise from the complex microcosm of the hospital environment. A clear understanding of this ever-evolving process, which includes frequent checking in with the staff and system as an in-built part of the process, is therefore key to a sustainable intervention and its implementation.

## Discussion

This systematic review of staff-reported barriers and facilitators to implementation of hospital-based, patient-focused interventions highlights two crucial pieces of information for researchers, policy-makers, and health service staff. First, there are key domains that must be considered to support effective implementation in hospital settings, and secondly, the interrelationships between these domains can be leveraged to address barriers and amplify facilitators. Our analysis indicated the presence of three overarching domains that could influence the implementation process: system, staff, and intervention. The evidence of distinct domains and their interrelationships confirms prior research and theory that implementation success is influenced by a dynamic range of barriers and facilitators. While the wide range of relevant sub-domains may seem overwhelming, it can also be empowering, as it highlights the many avenues through which researchers, health service staff, administrators, and managers can positively shape intervention design and implementation strategies. Each of the three main domains had a significant influence on implementation success; we discuss each in turn, describe interrelationships, and reflect on directions for future research below.

Barriers within the system domain confirmed the importance of understanding the broader organizational context, an issue that has been raised frequently in implementation research to date [56, 57]. The influence of these macro-level barriers was particularly evident in studies that described implementation across different hospital contexts [31, 58, 59]. These studies all showed that while the intervention design and processes were the same across sites, the cultures of each site were vastly different and faced their own unique barriers and enablers. Those interventions that responded to the hospital context and worked toward ease of integration were more likely to be reported as successful, in terms of adherence, acceptability, and sustainability [58]. Therefore, a thorough understanding of the system in which an intervention will be implemented can assist in intervention design. Several studies carried out barrier analyses relating to the organization prior to implementation, commonly using qualitative interviews or informal meetings. No studies identified in this review used validated measures for pre-assessment of organizational or staff level barriers. Recent research has generated a range of validated measures to assess organizational context including the Organizational Readiness for Implementing Change (ORIC) [60], the Organizational Readiness to Change Assessment (ORCA) [61] and Alberta Context Tool (ACT) [62]. Use of these measures, in conjunction with early-stage interviews and feedback from key stakeholders, may provide useful information on the context and highlight system level challenges that need to be addressed, potentially through intervention modification or tailored implementation strategies.

Barriers within the staff domain highlighted challenges at the micro level, including motivation toward change, personal beliefs regarding the intervention, understanding of the end-goals and outcomes, and level of skill and confidence. This demonstrates the need for implementation researchers to take the time to understand staff engagement and beliefs about the intervention and to generate specific strategies to address existing barriers. Studies in this review used a range of strategies to engage staff, including involvement in intervention development, targeted education and training to support and build confidence, and integration of ongoing feedback and regular contact to continually address concerns and provide a forum for staff to share experiences [5, 6, 44]. Recognizing staff as a dynamic and central factor in intervention design, implementation and maintenance is therefore likely to be crucial to ongoing sustainability.

Finally, intervention factors were consistently reported to play a strong role in implementation success. Almost every study named the barriers encountered in relation to the intervention itself. These were fairly consistent, with issues of ease of integration, face validity, safety/legality, and supportive strategies being commonly reported across the wide range of interventions that were reviewed. While much research in implementation science has focused on the contextual factors such as system and staff influences, recent research has highlighted this important role that intervention design plays in implementation processes [63]. Frameworks such as the CFIR [10] outline a range of facets within the intervention and its delivery process that should be considered, and our findings support this focus. Awareness of barriers is especially important in the design and deliver of complex, multi-faceted interventions, which are commonly implemented in hospital settings. Implementation of clinical pathways, patient-focused care initiatives, and evidence-based practice guidelines frequently engage multiple health disciplines and may demand that changes be made at the process and system levels in contrast to current practice. Implementing change can be demanding on staff and health services and interventions that are flexible and engage with needs of end users, are likely to produce better outcomes [57]. Therefore, researchers should consider intervention design and place more emphasis on pilot testing interventions to demonstrate feasibility and acceptability prior to full-scale implementation.

This review also provided novel insights into the associations between system, staff, and intervention domains, with each domain having possible influence on the others. Links between barriers across domains were more clearly recognized and more consistently addressed by those studies that reported using a theory or framework to guide their implementation [34]. This is likely due to the encouragement of iterative review and reflection that is central to most frameworks in the field. Interventions that had inbuilt flexibility, and allowed for ongoing change and tailoring, resulted in greater opportunities to introduce strategies and respond to unforeseen challenges. These new learnings can assist researchers, health service staff, administrators, and managers developing interventions to directly assess for challenges posed by context or culture and respond to this by tailoring their intervention where possible.

In undertaking this systematic review, we gave consideration to the relative benefits and detriments of inductive versus deductive analysis. Given the hospital context, and recognition of the systematic review as an iterative process [21], we elected to use an exploratory approach to remain open to the factors that may emerge from real-world studies within hospital settings. In line with our secondary aims, we recognized the breadth of determinant frameworks already exist and it was very useful to compare our findings within these frameworks, in order to explore similarities and differences. The three key domains identified in this review reinforce the use of theory-based frameworks to guide and support hospital-based implementation, as the factors outlined by such frameworks were clearly borne out in this real-world data. Our findings also contribute to the usefulness of existing frameworks, adding to the PARiHS framework by highlighting the important role of intervention factors, and to the CFIR by casting light on the associations between domains. Our domains showed significant overlap across the five domains of CFIR. However, it was challenging to decide where specific barriers from the studies we reviewed would best fit with pre-defined framework domains. For example, due to the limited information provided in some studies, it was unclear at times where a barrier would fit within the CFIR sub-domains; this applied in trying to determine the role of an individual involved in engagement, as studies did not always provide sufficient detail to code this barrier into an “opinion leader” versus a formally appointed “champion.” This type of fine grained differentiation may be of most relevance in situations where nuanced distinctions might influence the selection of implementation strategies at the development stage.

With five domains and 39 constructs, the CFIR provides a more nuanced conceptualization of factors impacting implementation success and therefore provides a means of expanding and exploring in more depth the domains identified in our analysis. In contrast, our review generated a simplified view of factors, which may be more pragmatic for busy hospital environments. In real world research, it is clear that at some points, pragmatism is required, while at other times, a more detailed understanding is needed, and this is a constant balance for implementation scientists.

We acknowledge that this review has some limitations. While every attempt was made to screen widely and inclusively, indexing studies in implementation is inconsistent and it is possible that some eligible studies were missed. Papers written in languages other than English were excluded, and 39 of the 43 studies were conducted in developed countries. Therefore, the findings outlined may be of less relevance to hospital-based implementation in developing nations. The quality of studies was variable, and in some cases involved very small sample sizes. The majority of studies collected qualitative data and at times did not provide significant detail about the interview methods or data analysis. Finally, in choosing to include only original research published in full, it is possible that we were unable to include some of the newest emerging research in the field (e.g., conference abstracts). There is significant debate about the exclusion of grey literature and unpublished research in systematic reviews, and it is noted that in choosing to exclude this research, there is a risk of publication bias in the findings presented [64].

Despite this, our review highlights knowledge gaps and areas for future study in the context of hospital-based implementation. Many studies published implementation results shortly after implementation, so questions about sustainability remain unanswered. This is supported by a recent scoping review by Tricco and colleagues, which showed that very few studies publish results about sustainability [65]. It is unclear whether the barriers and facilitators identified in this review will impact on long-term sustainability, and further research focused on the longer-term processes of change are warranted. Our review also noted significant variability in definitions of, and/or the outcomes used to assess, “implementation success” across different studies. This variability makes it hard to assess the generalizability of findings or to make broader comparisons across studies. A greater focus on outcomes with clearer definitions of successful implementation, such as the taxonomy proposed by Proctor et al. [19], would assist researchers to generate findings that can be more easily evaluated. In addition, while there has been a proliferation of studies focused on the introduction of new interventions in recent years, we found that a significant proportion of the papers identified in our initial search addressed the implementation process only anecdotally, without the collection of any formal data. The inclusion of formal assessments of the implementation process in future research will greatly add to the body of knowledge about the specific factors that influence successful translation of evidence into practice. Finally, the need to build flexibility into interventions emerged as a key facilitating factor. However, the balance between flexibility and fidelity is an ongoing challenge in the field. Cohen et al. [66] highlight the importance of clarity in research design and reporting regarding which elements of the intervention are adapted, to increase understanding of these processes within the readership. The StaRI guidelines suggest that this issue can be explored by differentiating between the core components of the intervention, to which fidelity is required, versus components or strategies that may be adapted by local sites to support effective implementation [18]. Adhering to the recommendations of these recent guidelines when reporting results will help to improve the quality of reporting and generating results that can be more clearly understood and used by others in the field.

## Conclusions

Our findings have clear practical implications for researchers and health service staff seeking to develop and implement feasible and acceptable interventions in hospital settings. They highlight the need to consider staff and system domains as active components in the change process rather than imposing change. An ongoing process of reflection and evaluation is indicated, with early engagement in intervention design, involvement and regular dialog with staff during pilot testing, and full-scale delivery of the intervention, including staff at administrative and managerial levels. Implementation scientists may benefit from reflecting on the interrelationships between the three domains identified in this review, to understand the bidirectional associations between different domains within the hospital setting. The greater our understanding of these associations, the more likely we are to be able to implement interventions that are meaningful, acceptable, and positively impact on health outcomes.

## Additional files

Additional file 1: Search terms by database. (DOCX 15 kb)

Additional file 2: Examples of excluded papers for each eligibility criterion. (DOCX 21 kb)

Additional file 3: Summary table of included papers. (DOCX 87 kb)
