# Supplementary material for: Hospital-based interventions: a systematic review of staff-reported barriers and facilitators to implementation processes
Source: Implement Sci. 2018 Feb 23;13:36. doi: 10.1186/s13012-018-0726-9 (PMC5824580; doi:10.1186/s13012-018-0726-9)
Supplement: Supplementary file 1 — Search terms by database. (DOCX 15 kb) [file 13012_2018_726_MOESM1_ESM.docx]

**Additional File 1. Search terms by database**

1. PsychInfo

(implementation$ OR dissemination$.mp OR exp Knowledge Transfer/ or "knowledge translation" OR roll-out$ OR rollout$ OR intervention$ or exp INTERVENTION/ OR exp Treatment Planning/ or treatment plan$ OR care plan$ OR pathway$ OR exp INNOVATION/ or innovation$ OR "complex intervention") AND (exp Health Care Services/ or exp Health Care Delivery/ or exp Health Care Utilization/ OR health service$ OR hospital service$ OR exp HOSPITALS/ or hospital$ OR health services research OR exp Health Care Services/ or clinical service$ OR hospital program$ OR or exp Hospital Programs/ OR tertiary service$) AND (facilitat$ OR barrier$ OR challenge$ OR challenging OR barrier analysis. OR "process analysis" OR enabl$ OR change agent) AND (psychosocial OR psychological OR psychology OR or exp HEALTH CARE PSYCHOLOGY/ or exp PSYCHOLOGY/ psychoncology)

1. Medline

(implementation$ OR or exp Health Plan Implementation/ OR dissemination$ OR "knowledge translation" OR or exp Translational Medical Research/ OR "knowledge transfer" OR rollout$ OR roll-out$ OR intervention$ OR treatment plan$ OR "care plan$" OR pathway$ OR innovation OR "complex intervention") AND (health service$ OR or exp Health Services/ OR exp "Delivery of Health Care"/ or health care services OR health care utilization OR exp Hospitals/ or hospital services$ OR or exp Hospitals, Public/ hospital$ OR or exp Hospitals/ OR health services research OR or exp Health Services Research/ clinical services$ OR hospital program$ OR hospitals, rural OR or exp Hospitals, Rural/ tertiary service$) AND (facilitat$ OR barrier$ OR challeng$ OR barrier analysis OR process analysis OR enabl$ OR change agent) AND (psychosocial OR psychological OR psychology OR or exp Psychology/)

1. Web of Science

(implementation OR roll-out OR effectiveness OR dissemination OR sustainability OR diffusion OR exchange OR innovation OR uptake)) AND (health services OR health care services OR health services research OR health care utilization OR health care delivery OR clinical services OR hospital services OR tertiary services)) AND (intervention OR treatment plan OR care plan OR pathway OR critical pathway OR complex intervention)) AND (barriers OR enablers OR facilitators OR challenges OR barrier analysis) AND (psychosocial OR mental health OR psychological)

1. Cinahl

(("implementation*" OR "roll-out*" OR (MH "Diffusion of Innovation") OR "dissemination$"OR "rollout$" OR "knowledge transfer" OR "knowledge translation" OR "intervention$" OR "treatment plan" OR (MH "Patient Care Plans+") OR"care plan$" OR "pathway$" OR (MH "Critical Path") OR "innovation$" OR "complex intervention")) AND (("health service$" OR (MH "Health Care Delivery+") OR "health care services" OR(MH "Hospital Programs") OR "hospital$" OR (MH "Health Services Research+") OR "health services research" OR "health care utilization" OR "facilitat*" (MH "Tertiary Health Care") OR "tertiary service*")) AND (("enabl*" OR"challeng*" OR "barrier*" OR "barrier analysis" "process analysis" OR "change agent" (MH "Change Management")) AND ((MH "Psychology+") OR "psychology" OR "psychological" OR "psychosocial")

1. Embase

(implementation$ OR "roll-out$" OR dissemination$ OR exp health care planning/ or "rollout$" OR exp translational research/ or knowledge translation OR intervention$ OR or exp intervention study/ exp treatment planning/ or "treatment plan$" OR "care plan$" OR exp clinical pathway/ or pathway$ OR innovation$ OR "complex intervention$" OR or exp intervention study/) AND ("health service$" OR or exp health service/ OR "hospital service$" OR or exp hospital service/ OR exp hospital utilization/ or hospital$ OR or exp hospital/ "health care service$" OR "health care delivery" OR or exp health care delivery/ exp health services research/ OR "clinical service$" OR "hospital program$" OR exp tertiary health care/ or "tertiary service$") AND (facilitat$ OR barrier$ OR challeng$ OR "barrier analysis" OR "process analysis" OR enabl$ OR "change agent$") AND (psychological OR psychosocial OR exp psychology/ or psychology )
