# Supplementary material for: Hospital-based interventions: a systematic review of staff-reported barriers and facilitators to implementation processes
Source: Implement Sci. 2018 Feb 23;13:36. doi: 10.1186/s13012-018-0726-9 (PMC5824580; doi:10.1186/s13012-018-0726-9)
Supplement: Supplementary file 2 — Examples of excluded papers for each eligibility criterion. (DOCX 21 kb) [file 13012_2018_726_MOESM2_ESM.docx]

**Additional File 2. Examples of excluded papers for each eligibility criterion**

| **Criterion** | **Exclusions in this area** | **Excluded paper example** | **Reason for exclusion** |
| --- | --- | --- | --- |
| **Original research published in full** | 1. Conference abstract, no full text accessible | Hudson P. Involving family caregivers in palliative care research: Challenges and strategies. Psychooncology. 2015;24:108. | Conference abstracts for which no full text could be accessed were excluded as there was insufficient detail to determine whether they met the additional exclusion criteria |
|  | 1. Book chapter | Camp CJ. Montessori-Based Dementia ProgrammingTM in Long-Term Care: A Case Study of Disseminating and Intervention for Persons with Dementia. Geropsychological interventions in long-term care. New York, NY: Springer Publishing Co; US; 2006. p. 295-314. | Book chapters were excluded as they reported on existing studies but did not contain any original research data not already published. |
|  | 1. Review paper | Whitaker, R., et al. Intervention now to eliminate repeat unintended pregnancy in teenagers (INTERUPT): A systematic review of intervention effectiveness and cost-effectiveness, and qualitative and realist synthesis of implementation factors and user engagement. Health Technology Assessment. 2016;20;16, 1-214. | Review papers were excluded as they summarized existing data but did not contain original research data. |
|  | 1. Commentary paper | Svikis DS, Reid-Quinones K. Screening and prevention of alcohol and drug use disorders in women. Obstetrics and Gynecology Clinics of North America. 2003;30:447-68. | Commentary papers were excluded as they did not contain original research data. |
|  | 1. Protocol | Bove DG, Overgaard D, Lomborg K, Lindhardt BO, Midtgaard J. Efficacy of a minimal home-based psychoeducative intervention versus usual care for managing anxiety and dyspnoea in patients with severe chronic obstructive pulmonary disease: a randomised controlled trial protocol. BMJ Open. 2015;5:e008031. | Protocol papers were excluded as they did not contain original research data. |
| **Hospital setting** | 1. School setting | Bell SG, Newcomer SF, Bachrach C, Borawski E, Jemmott JB, III, Morrison D, et al. Challenges in replicating interventions. Journal of Adolescent Health. 2007;40:514-20. | This paper assessed school-based health focused interventions, and was therefore excluded due to setting. |
|  | 1. Primary care setting | Bostrom AC, Schafer P, Dontje K, Pohl JM, Nagelkerk J, Cavanagh SJ. Electronic health record - Implementation across the Michigan Academic Consortium. Cin-Computers Informatics Nursing. 2006;24:44-52. | This paper assessed implementation in primary care settings, which were considered distinct to hospital settings and therefore excluded. |
|  | 1. Forensic setting | Segal A, Daffern M, Thomas S, Ferguson M. Needs and risks of patients in a state-wide inpatient forensic mental health population. Int J Ment Health Nurs. 2010;19:223-30. | This paper focused on research based in prison settings, and was therefore excluded by setting. |
|  | 1. Public health | Frieden TR, Henning KJ. Public health requirements for rapid progress in global health. Global Public Health. 2009;4:323-37. | This paper focused on global health changes, rather than a specific implementation based in a hospital health setting, and was therefore excluded. |
|  | 1. Population setting | Kirchner AT, Ladd DA, Elshaw JJ, Schlub JF. An inexpensive workplace initiative to motivate high-risk individual health improvement. Mil Med. 2013;178:e948-53. | This paper focused on implementation of a lifestyle intervention delivered in the workplace, which was considered distinct to hospital settings and therefore excluded. |
| **Population** | 1. Patient experiences of intervention | Ledderer L, Cour K, Hansen HP. Outcome of supportive talks in a hospital setting: Insights from cancer patients and their relatives. Patient. 2014;7:219-29. | This paper focused on patient experiences of the intervention, and did not collect any data on staff experiences of the implementation process. |
|  | 1. Government workers/Health policy makers | Reid G, Higgs P. Vietnam moves forward with harm reduction: An assessment of progress. Global Public Health. 2011;6:168-80. | This paper collected data on experiences of government workers carrying out health care reform, but the intervention was not hospital based, and the workers could not be classified as hospital staff. |
|  | 1. Community health workers | Leerlooijer JN, Kok G, Weyusya J, Bos AER, Ruiter RAC, Rijsdijk LE, et al. Applying Intervention Mapping to develop a community-based intervention aimed at improved psychological and social well-being of unmarried teenage mothers in Uganda. Health Educ Res. 2014;29:598-610. | This paper focused on community worker experiences, but the intervention was not hospital based, and the workers could not be classified as hospital staff. |
| **Interventions** | 1. IT intervention | Doran DM, Mylopoulos J, Kushniruk A, Nagle L, Laurie-Shaw B, Sidani S, et al. Evidence in the palm of your hand: Development of an outcomes-focused knowledge translation intervention. Worldviews Evid Based Nurs. 2007;4:69-77. | This intervention focused only on IT changes, and did not have any direct patient engagement or patient-focused outcomes. |
|  | 1. Medical Record intervention | McLane S. Designing an EMR planning process based on staff attitudes toward and opinions about computers in healthcare. Cin-Computers Informatics Nursing. 2005;23:85-92. | This intervention focused only on medical record changes, and did not have any direct patient engagement or patient-focused outcomes. |
|  | 1. Staff rostering intervention | Albertsen K, Garde AH, Nabe-Nielsen K, Hansen AM, Lund H, Hvid H. Work-life balance among shift workers: results from an intervention study about self-rostering. Int Arch Occup Environ Health. 2014;87:265-74. | These interventions were focused on staff administration outcomes, and did not have any direct patient focus. |
|  | 1. Management intervention, not patient care | Abuhejleh A, Dulaimi M, Ellahham S. Using lean management to leverage innovation in healthcare projects: Case study of a public hospital in the UAE. BMJ Innovations. 2016;2:22-32. | These interventions were focused on management and cultural changes, and did not have any direct patient focus. |
| **Formal data collection from participating staff about barriers and facilitators to the implementation process.** | 1. Intervention focused - Efficacy study | Berger JL. Incorporation of the tidal model into the interdisciplinary plan of care - a program quality improvement project. Journal of Psychiatric and Mental Health Nursing. 2006;13:464-7. | Data collected is only on outcomes related to the *intervention*, such as amount of staff who change their practice. No collection of any data regarding the implementation process, strategies or barrier analysis is reported. |
|  | 1. Intervention focused - Feasibility study | Charlton S, Muir L, Skinner TC, Walters L. Pilot evaluation of anterior dynamic ultrasound screening for developmental dysplasia of the hip in an Australian regional hospital. Rural and remote health. 2012;12:2091. | Data collected is focused on the feasibility of the *intervention*, based on staff and patient views of the intervention outcome data changes. No collection of any data regarding the implementation process, strategies or barrier analysis is provided. |
|  | 1. Intervention focused -Acceptability study | Caldon LJ, Collins KA, Reed MW, Sivell S, Austoker J, Clements AM, et al. Clinicians' concerns about decision support interventions for patients facing breast cancer surgery options: understanding the challenge of implementing shared decision-making. Health expectations: an international journal of public participation in health care and health policy. 2011;14:133-46. | Although the title suggests an implementation focus, data collected is focused on clinician attitudes toward the *intervention,* rather than how it would be implemented. No collection of any data regarding the implementation process, strategies or barrier analysis is provided. |
|  | 1. Implementation focused -Descriptive study | Anderson KL, Bruce SD. Putting your best foot forward in a challenging role: finding the resources needed to work in a freestanding radiation oncology clinic. Clin J Oncol Nurs. 2002;6:225-7. | This study describes the implementation process only from the point of view of the researchers, and this information is provided descriptively. No collection of any formal data (e.g. survey or interview) about the implementation process as it is experienced by staff involved. |
|  | 1. Implementation focused - Lessons learned study | Bond GE. Lessons learned from the implementation of a Web-based nursing intervention. Comput Inform Nurs. 2006;24:66-74. | This study describes the implementation process from the point of view of the researchers only. No collection of any formal data (e.g. survey or interview) about the implementation process as it is experienced by staff involved. |
