# Supplementary material for: Hospital-based interventions: a systematic review of staff-reported barriers and facilitators to implementation processes
Source: Implement Sci. 2018 Feb 23;13:36. doi: 10.1186/s13012-018-0726-9 (PMC5824580; doi:10.1186/s13012-018-0726-9)
Supplement: Supplementary file 3 — Summary table of included papers. (DOCX 87 kb) [file 13012_2018_726_MOESM3_ESM.docx]

**Additional File 3. Summary table of included papers**

| **Author** | **Title** | **Year** | **Data type^1^** | **Key barriers** | **Key facilitators** |
| --- | --- | --- | --- | --- | --- |
| Belkora, J. K., et al.[50] | Monitoring the implementation of Consultation Planning, Recording, and Summarizing in a breast care center | 2008 | Qualitative | Time; organisation of implementation; disruption to normal workflow; resources | Modify intervention based on staff feedback; seek feedback and audit; streamline components of intervention |
| Bergh, A.M., et al. [39] | Progress with the implementation of kangaroo mother care in four regions in Ghana | 2013 | Qualitative | Lack of structure for referral process; poor functioning of feedback network; patient related barriers; cultural influences; lack of clear document/policy | Structural, system and personnel factors: support from management, communication at all levels; demonstrated leadership by co-ordinators, access to continued education; input from wide range of health care works, efficient functioning of steering committee; awareness strategies (e.g. posters) |
| Blake, S.C., et al.[44] | Facilitators and barriers to 10 national quality forum safe practices | 2006 | Qualitative | Resistance to change; fear and mistrust regarding the implementation/intervention process; poor communication | Education, reinforcement and positive incentives; audit and feedback; leadership support; champions; engaging staff buy-in; external requirement pressures; strategies to improve communication/collaboration; training and team work |
| Boscart, V. M., et al. [67] | Using psychological theory to inform methods to optimize the implementation of a hand hygiene intervention | 2012 | Qualitative | Skill level; awareness/focus on intervention; time/resources; competing priorities; inter-group conflict; overload/fatigue | Professional identity/role; education/training; environmental factors; motivation/commitment; social support/collaboration; reminders |
| Bradford, A. N., et al. [66] | Barriers to Implementation of a Hospital-Based Program for Survivors of Traumatic Injury | 2013 | Mixed methods | Time; lack of dedicated funding; lack of institutional mandate to implement; administrative challenges; clinical concerns; poor communication; technical difficulties; lack of information/skill | Provision of evidence regarding outcomes; champion driven approach or top-down approach both can lead to effective implementation; increasing and expand training programs; improved follow-up guidance |
| Cheyne, H., et al. [59] | Empowering change: Realist evaluation of a Scottish Government programme to support normal birth | 2013 | Qualitative | Resistant context; staff mindset; medical culture; communication; lack of training and confidence; lack of motivation | Fit with existing system; familiarity with processes; management support; positive mindset; |
| Clark, J. B., et al. [36] | Staff Perceptions of End-of-Life Care following Implementation of the Liverpool Care Pathway for the Dying Patient in the Acute Care Setting: A New Zealand perspective | 2012 | Qualitative | Time/workload; physical environment limitations; physician attitudes; changes in direction of care causing delays ; lack of communication skills; workflow | Improving knowledge and confidence; ongoing education/training; support from specialist services; staff commitment |
| Clement, C. M., et al. [54] | Perceived facilitators and barriers to clinical clearance of the cervical spine by emergency department nurses: A major step towards changing practice in the emergency department." | 2011 | Quantitative | Workload; legal concerns; professional role; time, safety concerns; lack of fit to patient; too difficult; lack of skill/ understanding of intervention; negative attitude | Good support from physicians, nurse educators and managers; peer support/cooperation; refresher sessions; visual aids; local champions |
| Cranwell, K., et al.[20] | Improving mental health service users' with medical co-morbidity transition between tertiary medical hospital and primary care services: a qualitative study | 2016 | Qualitative | Challenges related to access pathways, communication and continuity of care, clinician attitudes and lack of opportunity for caregiver involvement | Clinician expertise; engagement and accessibility |
| Dubenske, L. L., et al. [51] | Development and implementation of a clinician reporting system for advanced stage cancer: initial lessons learned. | 2008 | Qualitative | Functionality of tool; limited use by patient; privacy concern; limits of the technology; difficulties with measurement tools | Responding to clinician feedback regarding the tool; altering forms to address privacy concerns; altering measurement to increase detail; customisable system; enhancing fit with existing processes |
| Dudgeon, D., et al. [52] | Cancer Care Ontario's experience with implementation of routine physical and psychological symptom distress screening. | 2012 | Qualitative | Process issues including lack of consensus and guidance on screening tools; technology failures; resource constraints; burden on staff; resistant culture | Centralized project management; a dedicated person to oversee implementation; regional engagement; clear aims; regular reporting/data analysis; implementation of quality improvement methodologies; senior leadership and champions; flexibility |
| Elwell, L., et al. [47] | Challenges of implementing routine health behavior change support in a children's hospital setting | 2014 | Qualitative | Health professional knowledge, beliefs and behaviours; roles and responsibilities; confidence in skills; difficulties in patient engagement; lack of knowledge | increase knowledge and skills, access to training; support staff to overcome patient related barriers ; fit with personal beliefs and role responsibility |
| Francis, J., et al. [49] | Selective decontamination of the digestive tract in critically ill patients treated in intensive care units: a mixed-methods feasibility study (the SuDDICU study). | 2014 | Qualitative | Difficulty/complexity of intervention; multiple clinicians and contexts; workload/time; cost; staff changes; resources | Becomes part of routine; perceived effectiveness of intervention; fit with existing routine; part of policy; peer support |
| Goldenhar, L. M., et al. [68] | Huddling for high reliability and situation awareness | 2013 | Qualitative | Time and resources | Training and coaching; dedicated time of day; scheduled venue; accountability and collaboration |
| Hogan, D. L. & Logan, J. [69] | The Ottawa Model of Research Use: a guide to clinical innovation in the NICU | 2004 | Mixed methods | Practice setting; competing priorities; heavy workload; stress, lack of skill/confidence; not suited to all groups | Simplicity of intervention; fit with existing practices; increase training and knowledge/skills |
| Hsu, C., et al. [53] | Incorporating Patient Decision Aids into Standard Clinical Practice in an Integrated Delivery System." | 2013 | Qualitative | Negative attitudes; lack of engagement; concern about patient impact; difficulty identifying suitable patients; time; resources; lack of formal leadership; hard to manage timing of intervention | Higher level support; clear system and feedback; engagement of end-users in implementation development and process; finding ways to address staff concerns |
| Hughes, R., et al.[31] | "It just didn't work: the realities of quality assessment in the English health care context." | 2004 | Qualitative | Time; paperwork; competence/confidence; staff shortages; patient concerns; ethical concerns; eligibility requirements; staff beliefs regarding validity | Staff enthusiasm and interest; positive outcomes for patients and staff; close relationships with patients |
| Jones, A. [30] | Implementation of hospital care pathways for patients with schizophrenia." | 2000 | Qualitative | Site/structural changes; poor morale/engagement; poor documentation; lack of permanent staff; lack of support from staff and facilitator; new professional role boundaries; too simplistic to reflect the complexity of care | Additional training to target increased agency staff |
| Kahan, D., et al.[33] | Integrating care for frequent users of emergency departments: implementation evaluation of a brief multi-organizational intensive case management intervention | 2016 | Qualitative | Low referral rates; challenges in management of multi-organisational initiative; variable adherence; lack of access to psychiatric resources; lack of dedicated staff and limited local system capacity; insufficient training and technical assistance | Service delivery factors- organisational capacity and history of collaborative relationships; support system factors (e.g. training and supervision) |
| Kane, J. C., et al. [29] | Challenges for the implementation of World Health Organization guidelines for acute stress, PTSD, and bereavement: a qualitative study in Uganda | 2016 | Qualitative | Lack of training; poor staff/patient ratio; patient related barriers; provider beliefs; cultural acceptability; patient contextual factors; structural barriers (resource limitations, management and communication problems; transport issues) | Training and capacity building; additional staff; resources allocation and task sharing; flexibility within intervention; cultural adaption; prioritise staff well-being; modification of components of intervention |
| Karlsson, A., et al. [43] | Feasibility of a computerized alcohol screening and personalized written advice in the ED: opportunities and obstacles. | 2005 | Mixed methods | Stress; workload; times; negative attitude; concerns re liability; role fit; lack of current guideline; concern about patient reaction; physical location issues; workflow disruption; lack of awareness/recall | Provision of guidelines; training |
| Kirk, J. W., et al. [38] | Barriers and facilitators for implementing a new screening tool in an emergency department: A qualitative study applying the Theoretical Domains Framework | 2016 | Qualitative | Professional role/identity - culture and role boundaries within this; beliefs about consequences regarding time and threat to professional identity | Meaning and sense-making of intervention purpose and consequences; leadership; resources |
| Lee, A. K., et al. [70] | Perceived facilitators and barriers to the implementation of an advanced practice: nursing intervention for HIV regimen adherence among the seriously mentally ill. | 2006 | Qualitative | Time for implementation; time taken to adapt to protocol and locate clients | Relationship with patient, ability to take the time needed |
| Liisa, A. A., et al. [40] | Health care personnel's experiences of a bereavement follow-up intervention for grieving parents. | 2011 | Qualitative | Lack of expertise; lack of fit with role; lack of supervision; lack of shared collaboration; negative patient attitudes; competing priorities ; difficulties with information transfer | Positive staff attitude; communication; engagement/desire to help; supportive organisational conditions including training, supervision, peer support and resources; patients receptivity |
| Martin, M. L., et al. [71] | Integrating an evidenced-based research intervention in the discharge of mental health clients | 2007 | Qualitative | Roles/responsibilities; communication and staff relationships; values and beliefs of staff/clients/community; lack of resources; processes of care | Support from colleagues; practice/policy/education |
| McAteer, J., et al.[27] | Using psychological theory to understand the challenges facing staff delivering a ward-led intervention to increase hand hygiene behavior: a qualitative study | 2014 | Qualitative | Time, lack of staff; perceived negativity from other staff; stress | Confidence and skill level; understanding and motivation; implementation consistent with existing role |
| Mello, M. J., et al. [55] | Translation of alcohol screening and brief intervention guidelines to pediatric trauma centers | 2013 | Qualitative | Insufficient training; electronic record issues; lack of staff support; staffing changes; incomplete screening; competing priorities; changes in site leader; eligibility confusion | Effort led by trauma co-ordinator; strong use of electronic records; strong partnering with social work; real-time monitoring to assure completion of intervention |
| Melnyk, B. M., et al. [48] | Translating the evidence-based NICU COPE program for parents of premature infants into clinical practice: impact on nurses' evidence-based practice and lessons learned | 2010 | Qualitative | Competing priorities; structural/environmental changes (ward renovation); time | Mentors; provision of support; reminders and visual cues |
| Moody, G., et al. [34] | An action research approach to the development of a clinical pathway for women requiring Caesarean sections | 2001 | Qualitative | Lack of awareness; need for further training; some lack of staff compliance; variation required for some units (e.g. NICU) | Ongoing feedback from all users, with continual modifications to intervention as required; regular in-services and updates |
| Nithianandan, N., et al.[28] | Factors affecting implementation of perinatal mental health screening in women of refugee background | 2016 | Qualitative | Skills/abilities; lack of role clarity; fragmented communication; lack of time/resources; staff beliefs about consequences; environmental context; lack of continuity of care | Fit with sense of professional identity ; social support ; increase continuity of care; clear referral guidelines (e.g. flowcharts; clear documentation and communication |
| Nollen, C., Drainoni, M. L. & Sharp, V.[32] | Designing and delivering a prevention project within an HIV treatment setting: lessons learned from a specialist model | 2007 | Qualitative | Patient engagement and retention; beliefs regarding intervention and how it would function; staff workload, time, skills and motivation | Alterations to support patient engagement; reminders, incentives, dedicated research co-ordinator; training |
| Oestrich, I., Austin, S. & Tarrier, N.[46] | Conducting research in everyday psychiatric settings: Identifying the challenges to meaningful evaluation | 2007 | Qualitative | Staff lack of experience and motivation; shiftwork; organisational context factors; communication; environmental setting; staff turnover/leave; patient characteristics | Train staff in engagement; additional support provided; increase timeframe and flexibility of intervention; information provided on role and benefits of intervention; ongoing feedback; audit of skills and time; use of existing organisational communication systems; success stories; collaborative research approach |
| Pace, K., et al. [45] | Barriers to successful implementation of a clinical pathway for CHF | 2002 | Qualitative | Procedural inconsistences; lack of awareness, lack of feedback and education, complexity of intervention; increased work burden; beliefs re perceived need for intervention; poor fit to population; role flexibility and identity; lack of fit with existing system; poor communication | Assessment of patient population needs; assessment of work culture issues; adapt intervention components; intensive communication and education; monitoring of outcome indicators |
| Parker, C., et al. [72] | Tailored tobacco dependence support for mental health patients: a model for inpatient and community services | 2012 | Qualitative | System policy and procedures; lack of resources; lack of clear targets; insufficient training; lack of knowledge and negative staff attitude; patient factors related to illness complexity | Comprehensive staff training; dissemination of audit results; development of instruments and pathways; liaison with management; flexibly response to patient needs |
| Passalqua, R., et al. [73] | Feasibility of a quality improvement strategy integrating psychosocial care into 28 medical cancer centers (HuCare project) | 2016 | Quantitative | Lack of resources (staff, facilities, funds); excessive workload; lack of confidence in intervention effectiveness and own abilities; lack of management support; lack of economic incentive; poor motivation; perceived external interference | Numerous editions of courses offered in easily accessible locations; simulation of question prompts included in training; require centres to outline their own screening strategy |
| Rankin, N. M., et al. [6] | Everybody wants it done but nobody wants to do it: an exploration of the barrier and enablers of critical components towards creating a clinical pathway for anxiety and depression in cancer | 2015 | Qualitative | Lack of time; lack of qualified staff; concerns regarding duty of care and ethical responsibilities; lack of role clarity; patient reluctance; health professional fatigue with interventions | Ownership/engagement at the team level; support from leadership and management; integration into existing hospital system; intervention incorporated into policy; clarity on role responsibilities; education and training; evidence base regarding benefits; integration/communication across health services |
| Ross, F., O'Tuathail, C. & Stubberfield, D.[42] | Towards multidisciplinary assessment of older people: exploring the change process | 2005 | Qualitative | Communication across services; high workload and staff vacancies; variation in practice; fragmented decision-making processes; perceived loss of expertise; lack of role clarity | Regular staff engagement and trust; ownership by staff; shared language; flexibility in tailoring intervention; access to additional resources; focused training |
| Schmied, V., et al. [37] | Ten steps or climbing a mountain: a study of Australian health professionals' perceptions of implementing the Baby Friendly Health Initiative to protect, promote and support breastfeeding | 2011 | Qualitative | Time; institutional barriers; lack of compliance; misinterpretation of intervention; workload; time; conflict with institutional priorities; staff using 'short cuts' to combat lack of time | Belief and commitment; awareness of evidence and positive outcomes to patients and community; benefits to staff |
| Smid M., et al. [41] | Bringing two worlds together: Exploring the integration of traditional midwives as doulas in Mexican public hospitals | 2010 | Qualitative | Role responsibilities and perceptions about working with other disciplines; structural barriers (space and transport); cultural barriers; lack of confidence | Interactive training; focus on improving professional relationships; focus on benefits to all staff; clear role definition |
| Sorsdahl, K., et al. [35] | Screening and brief interventions for substance use in emergency departments in the Western Cape province of South Africa: views of health care professionals | 2014 | Qualitative | Clinic level: poor fit with current system; lack of private space, high workload and competing priorities. Patient level: challenges in overcoming aggression, engagement and attendance | Staff had a strong perceived need for the intervention; clear timing and schedule for intervention |
| Strauss, S. M., et al.[74] | Barriers and Facilitators in Implementing "Prevention for Positives'' Alcohol-Reduction Support: The Perspectives of Directors and Providers in Hospital-Based HIV Care Centers | 2012 | Qualitative | Time; patient lack of disclosure; staff doubt about utility/need for screening; limited sense of efficacy; organisational systems; provider specialisation means that most staff did not address these issues | having adequate time for intervention; having specific tools and points to inform patients; modification to intervention (shorter tool) |
| Van OsMendorp, H., et al. [58] | The tailored implementation of the nursing programme 'Coping with itch' | 2008 | Quantitative | Lack of financial incentive; time; lack of patient or staff co-operation; lack of recall/knowledge; staff resistance; lack of perceived benefit; lack of managerial support; poor fit with existing work style | Creation of new structures/spaces; education and training; peer review; creation of opinion leaders; multidisciplinary meetings; use of awareness-raising activities (posters etc.) |
| Wajanga, B. M. K., et al.[5] | Healthcare Worker Perceived Barriers to Early Initiation of Antiretroviral and Tuberculosis Therapy among Tanzanian Inpatients | 2014 | Qualitative | Lack of resources; poor integration between services; lack of staff to complete intervention; lack of awareness/acceptance of new guidelines; intervention too complex/involved; lack of patient disclosure | Increase availability of resources; train additional staff to perform new roles; build flexibility into intervention; create responsible 'on-call team'; continuing education; cultivation of positive multidisciplinary team approach and partnerships |

1. Data type refers to the methodology used by the study to collect *only* the data on barriers and facilitators specifically
